# Changes in Local Atrial Electrograms and Surface ECG Induced by Acute Atrial Myocardial Infarction

**DOI:** 10.3389/fphys.2020.00264

**Published:** 2020-04-17

**Authors:** Gerard Amorós-Figueras, Elena Roselló-Diez, Damian Sanchez-Quintana, Sergi Casabella-Ramon, Esther Jorge, Jorge Nevado-Medina, Dabit Arzamendi, Xavier Millán, Concepción Alonso-Martin, Jose M. Guerra, Juan Cinca

**Affiliations:** ^1^Department of Cardiology, Hospital de la Santa Creu i Sant Pau, Institute of Biomedical Research IIB Sant Pau, CIBERCV, Universitat Autònoma de Barcelona, Barcelona, Spain; ^2^Department of Cardiac Surgery, Hospital de la Santa Creu i Sant Pau, Institute of Biomedical Research IIB Sant Pau, Universitat Autònoma de Barcelona, Barcelona, Spain; ^3^Department of Anatomy and Cell Biology, Faculty of Medicine, University of Extremadura, Badajoz, Spain

**Keywords:** atrium, ischemia, ECG, electrophysiology mapping, histopathology

## Abstract

**Background:**

Atrial coronary branch occlusion is a hardly recognizable clinical entity that can promote atrial fibrillation. The low diagnostic accuracy of the ECG could deal with the characteristics of the ischemia-induced changes in local atrial electrograms, but these have not been described.

**Objectives:**

We analyzed the effects of selective acute atrial branch occlusion on local myocardial structure, atrial electrograms, and surface ECG in an experimental model close to human cardiac anatomy and electrophysiology.

**Methods:**

Six anesthetized open-chest anesthetized pigs underwent surgical occlusion of an atrial coronary branch arising from the right coronary artery during 4 h. Atrial electrograms and ECG were simultaneously recorded. One additional pig acted as sham control. In all cases, the hearts were processed for anatomopathological analysis.

**Results:**

Atrial branch occlusion induced patchy atrial necrosis with sharp border zone. During the first 30 min of occlusion, atrial electrograms showed progressive R wave enlargement (1.8 ± 0.6 mV vs. 2.5 ± 1.1 mV, *p* < 0.01), delayed local activation times (28.5 ± 8.9 ms vs. 36.1 ± 16.4 ms, *p* < 0.01), ST segment elevation (−0.3 ± 0.3 mV vs. 1.0 ± 1.0 mV, *p* < 0.01), and presence of monophasic potentials. Atrial ST segment elevation decreased after 2 h of occlusion. The electrical border zone was ∼1 mm and expanded over time. After 2 h of occlusion, the ECG showed a decrease in P wave amplitude (from 0.09 ± 0.04 mV to 0.05 ± 0.04 mV after 165 min occlusion, *p* < 0.05) and duration (64.4 ± 8.0 ms vs. 80.9 ± 12.6 ms, *p* < 0.01).

**Conclusion:**

Selective atrial branch occlusion induces patchy atrial infarction and characteristic changes in atrial activation, R/S wave, and ST segment that are not discernible at the ECG. Only indirect changes in P wave amplitude and duration were appreciated in advanced stages of acute coronary occlusion.

## Introduction

Atrial myocardial infarction is a potentially harmful disease that can provoke atrial mechanical depression, systemic embolization, atrial arrhythmias, and cardiac rupture (Lazar et al., 1988). It is caused by occlusion of the atrial coronary branches ordinarily emerging from the proximal segments of the right (RCA) or the left circumflex (LCX) coronary arteries (Baroldi et al., 1956; James and Burch, 1958). The diagnosis is often established at autopsy in association with ventricular myocardial infarction. In a series of 182 necropsy cases of myocardial infarction, the incidence of atrial infarction was 17% (Cushing et al., 1942).

Selective occlusion of an atrial coronary branch may result from a rupture of a local atherosclerotic plaque or, accidentally, during percutaneous coronary interventions (Álvarez-García et al., 2013, 2016). This condition predisposes to arrhythmia reentry as evidenced in the canine model (Sinno et al., 2003; Nishida et al., 2011) but is hardly recognized at the surface ECG. The diagnostic paucity of the ECG could be due to intrinsic characteristics of the conventional recording systems or to a weak impact of atrial branch occlusion on local myocardial structure and electrophysiology.

In the clinical practice, the ECG features that allow suspecting atrial myocardial ischemia are changes in P wave morphology and PR-segment deviation (Lazar et al., 1988; Lu et al., 2016; Yıldız et al., 2018) although these are inconstant and can require a reference ECG to be reliably discerned. Changes in the QRS complex and ST segment of local electrograms are specific markers of acute myocardial ischemia in the ventricles (Janse et al., 1979), and theoretically, similar electrical changes should be found in the atrial electrograms during atrial branch occlusion, but it has not yet been proven.

Therefore, this study aimed to analyze the effects of selective occlusion of an atrial coronary branch on atrial electrograms and surface ECG in a model close to human cardiac coronary distribution and electrophysiology.

## Materials and Methods

### Study Population

This study involved seven anesthetized domestic swine (Landrace-Large White cross) weighing 40 ± 5 kg (six successful animals submitted to 4 h ischemia and one sham animal). The animals were premedicated with midazolam (0.6 mg/kg) and ketamine (12 mg/kg) intramuscularly. General anesthesia was induced with intravenous propofol (2–4 mg/kg) and was maintained with a mixture of oxygen and sevoflurane inhalation (2.5–3.5%). After endotracheal intubation, they were mechanically ventilated. Analgesia was maintained during the entire procedure with intravenous fentanyl (2.5 μg/kg). The study protocol was approved by the Animal Care and Use Committee of our institution and fully conformed the Guide for the Care and Use of Laboratory Animals, 8th edition (National Research Council, 2010).

### Experimental Procedures

#### Atrial Coronary Branch Occlusion

**FIGURE 1:**
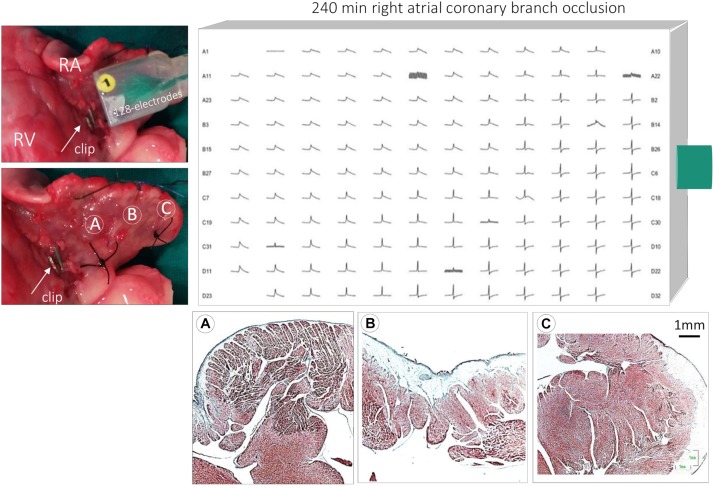
Illustration of the experimental preparation. Upper left: photograph of an extracted porcine heart showing the placement of the 128-electrode patch on the right atrial epicardium. The arrow shows an hemostatic clip occluding an atrial coronary branch from the right coronary artery. Encircled letters A, B, and C indicate atrial regions extending from the closest occlusion site to the remote sites of the atrial appendage. Upper right: local atrial electrograms recorded with a 128-electrode patch 240 min after occlusion of the right atrial coronary branch. Bottom: histological microphotographs of the right atrium showing transmural infarction in region A, subendocardial in region B, and normal myocardium in regions C. RA, right atrium; RV, right ventricle.

A median sternotomy was performed, and the pericardium was opened to expose the heart in a pericardial cradle. Atrial coronary branches arising from the right coronary artery were identified by fine direct dissection and separated from the surrounding fat tissue. In all cases, the atrial coronary branches originated at the proximal segment of the right coronary artery. Then, the atrial coronary branch was selectively occluded with hemostatic clips (Peters Surgical, France) (Figure 1) during 4 h in six pigs. One additional pig was submitted to the same experimental procedures, but the atrial coronary branches were not occluded (sham experiment). The thorax was maintained opened during the 4 h of atrial branch occlusion.

#### Atrial Epicardial Mapping

The epicardial unipolar atrial electrograms (Figure 1) were recorded with 128 electrodes spaced 1 mm mounted on a patch of 17 mm × 12.5 mm. This probe was gently sutured with Prolene 3/0 snare (Ethicon) to the atrial epicardium in a region close to the isolated atrial coronary branch. The reference electrode was placed at the upper mediastinal region close to the thymus and to the upper border of the sternotomy. Within this discrete anatomical region, we chose the site that provided well voltage and stable atrial electrograms. Then, the position of the reference electrode remained unaltered during the experiment. The local electrical potentials were recorded with a BioSemi acquisition system (BioSemi, Netherlands). Recordings were obtained at a sampling rate of 2 kHz and stored continuously during the first 60 min of atrial coronary occlusion and, thereafter, every 15 min until completing the 4-h occlusion. An offline analysis of the atrial electrograms was done with a custom-made Matlab script (MathWorks, United States), and the following parameters were measured: R/S wave amplitude, local activation time, measured between the start of increment of the R wave and at the highest downstroke slope after the R wave peak, and ST segment deviation from the J point. To have a quantitative appreciation of the changes in these parameters, we selected a group of six neighboring electrodes with an appropriate signal/noise ratio that have shown monophasic potentials. We defined as monophasic potentials all electrograms showing increased R wave amplitude together with loss of S wave and appearance of ST segment elevation.

#### Surface Electrocardiogram

The ECG was continuously recorded using a CardioSoft system (GE Healthcare, Germany), as previously reported (Noriega et al., 2013). The ECG was recorded at baseline and continuously during atrial coronary artery occlusion at a sampling rate of 200 Hz. All ECG recordings analyzed in this study were obtained in the open chest condition. Changes in the amplitude and duration of the P wave, QRS-complex duration, and ST segment at the J point were measured with Matlab scripts at ECG lead II using signal averaging in a window time of 1 min. We selected lead II because, in clinical practice, it is commonly used to evaluate the P wave characteristics, and moreover, the signal voltage of the lead was less affected after the median sternotomy.

#### Anatomopathological Examination

After 4 h of atrial coronary occlusion, pigs were euthanized by electrically inducing ventricular fibrillation. The heart was explanted with the 128 electrodes sutured in its initial position. Then, the position of the 128-electrode patch was marked with fine Prolene snares sutured at each electrode corner (Supplementary Figure S1). This procedure was done to evaluate a potential correspondence between the electrical electrograms and the underlying anatomical substrate. The heart was then cleaned from blood and fixed in buffered formalin. The atria were separated from the ventricles and embedded in a paraffin block for histological analysis. Sections of 12 μm were mounted and stained with Masson’s trichrome. A morphometry study was performed in six infarcted pigs to evaluate the average extent of myocyte degeneration (myolysis). As described previously (Climent et al., 2004), digital images were taken of the sections stained with Masson’s using a Nikon SMZ 1500 microscope (Nikon, United States). Morphometry was performed at least in four fields per atrial sample, in the regions of maximum extent of infarcted area, using a grid covering an area of ∼4 mm^2^ of atrial sample. The areas of myolysis were expressed as a percentage of the entire limits of the grid. Blood vessels, perivascular interstitial tissue, and intertrabecular spaces between the pectinate muscles were excluded from the morphometric analysis. The identification of the areas of myolysis was done by direct visualization of the histological preparation by an expert anatomopathologist at proper magnification.

### Data Analysis

Quantitative data were expressed as the mean ± standard deviation (SD). The ordinary two-way ANOVA test with Dunnett’s multiple comparison correction was used to evaluate the significance of the changes from baseline to selected occlusion times in the atrial R/S waves, local activation time, and ST segment. The ANOVA test was also used to assess the significance of the evolving changes in P wave morphology, QRS duration, and ST segment displacement in the ECG. The paired *t*-test was used to assess the significance of the changes in the number of electrodes showing ischemic changes. A *p* < 0.05 was considered significant. All analyses were performed using SPSS v.22.0 (IBM-SPSS, United States).

## Results

### Anatomopathological Findings

**FIGURE 2:**
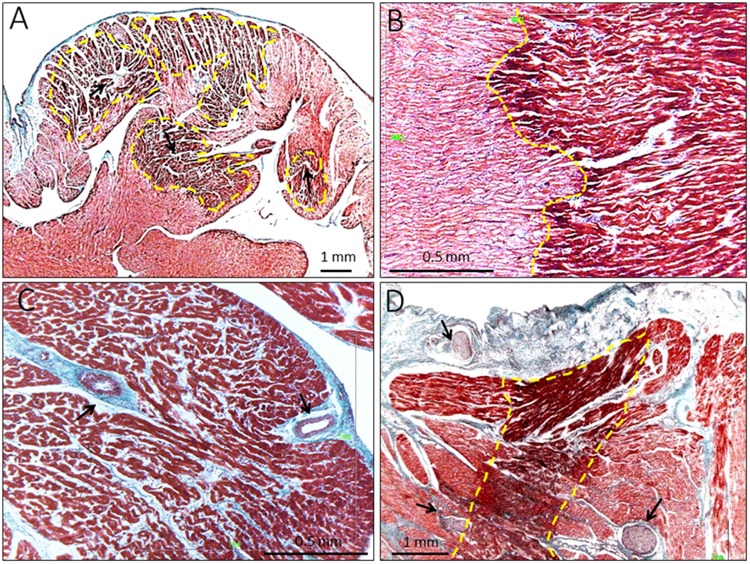
Histological findings in pigs submitted to 4-h of atrial coronary branch occlusion. **(A)** Microphotograph showing areas of “patchy” necrosis with myocardial fiber disarray (arrows) and sharp border zone (yellow marks). **(B)** Detailed amplification of the ischemic border zone showing a sharp band of interdigitating ischemic and normal cells (yellow marks). **(C)** Subendocardial infarction involving a pectinate muscle and small arterioles (arrows). **(D)** Necrotic foci (yellow marks) in the vicinity local atrial epicardial neural fibers (arrows).

As illustrated in Figure 2A, occlusion of an atrial coronary branch induced degeneration and coagulative necrosis of groups of cardiomyocytes. The necrosis depicted a “patchy” distribution and showed myocardial fiber disarray with cellular infiltration. The transitional ischemic border zone was abrupt and was composed of an interdigitating band of normal and ischemic cells (Figure 2B). The infarction affected subendocardial regions of the pectinate muscles and encompassed small arterioles (Figure 2C). In some instances, the necrotic foci appeared near local atrial epicardial or intramyocardial neural fibers (Figure 2D). The morphometric analysis in the sections showing the greatest infarct extent revealed that the mean area of myolysis in the six pigs was ∼54% (range, 37.7–77.8%) of the entire examination field. Supplementary Figure S2 illustrates representative examples of the measured areas of myolysis. The atria of the sham-operated pig were free of myocardial necrosis.

### Atrial Epicardial Mapping

**FIGURE 3:**
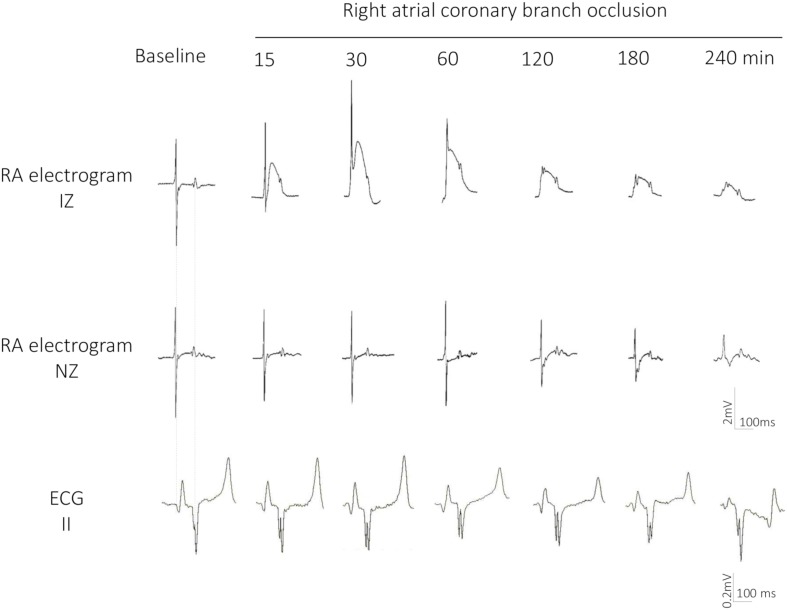
Evolving changes in epicardial atrial electrograms and surface ECG lead II in a pig submitted to 4 h of atrial coronary branch occlusion. Local electrograms in an ischemic (IZ) and normal zone (NZ) recorded at baseline and after 15, 30, 60, 120, 180, and 240 min of right atrial coronary branch occlusion showing increase in R wave amplitude, disappearance of the S wave, ST segment elevation, and monophasic potentials.

At baseline conditions, the atrial electrograms showed an R/S complex (Figure 3) with a duration of 48.6 ± 10.2 ms, closely followed by isoelectric ST segment. Figure 3 also illustrates the time correspondence between the local atrial signals and the surface ECG: the atrial R/S complex coincided with the surface P wave and the atrial ST segment with the PR segment of the ECG.

**FIGURE 4:**
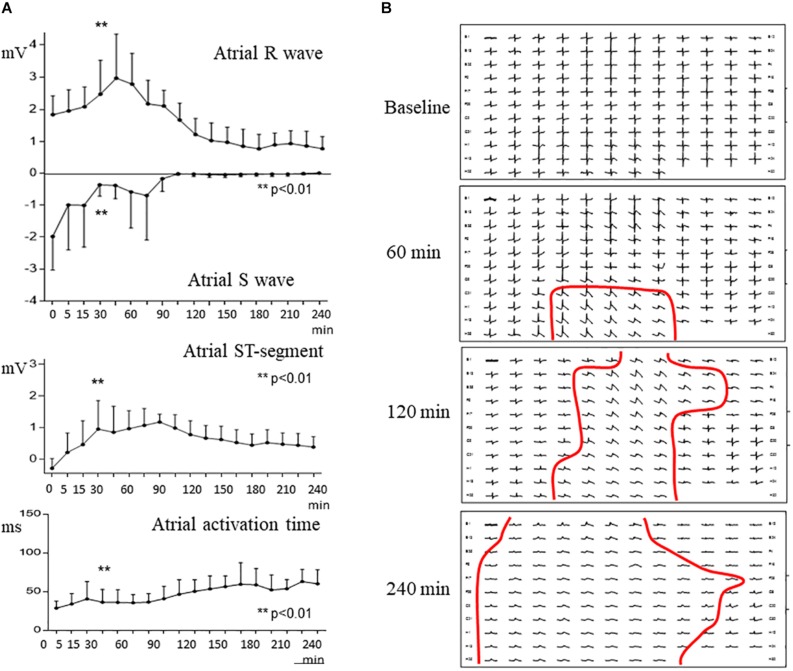
Evolving changes in atrial epicardial mapping with 128 electrodes in a pig submitted to 4 h of atrial coronary branch occlusion. **(A)** Mean values of atrialR wave, S wave, ST segment, and activation time in selected electrograms at baseline and at specific times after atrial coronary branch occlusion. **(B)** Mapping of epicardial atrial unipolar electrograms at baseline, 60, 120, and 240 min after atrial coronary branch occlusion. Red lines delineate the electrical border zone encompassing one or two electrodes. **p < 0.01; from 0 to 165 min of ischemia.

During the first 30 min of atrial branch occlusion (Figures 3, 4), the atrial epicardial electrodes overlying the ischemic area showed the following: (a) progressive increase in the R wave amplitude (from 1.8 ± 0.6 mV at baseline to 2.5 ± 1.1 mV at 30 min occlusion, *p* < 0.01), (b) disappearance of the S wave (from −2.0 ± 1.1 mV to −0.4 ± 0.4 mV, *p* < 0.01), (c) prolongation of local activation time (from 28.5 ± 8.9 ms to 36.1 ± 16.4 ms, *p* < 0.01), and (d) gradual elevation of the ST segment leading to monophasic potentials (from −0.3 ± 0.3 mV to 1.0 ± 1.0 mV, *p* < 0.01). After 30–40 min of occlusion, the affected atrial electrograms depicted a progressive decline in R wave amplitude and ST segment elevation maximal at 4 h of occlusion (R wave, 0.8 ± 0.4 mV; ST segment, 0.4 ± 0.3 mV; *p* < 0.01, from baseline). Electrodes not showing ST segment changes depicted a reduction in R wave amplitude of ∼30% after 3 h of occlusion (Figure 4A). In two cases, we observed a transient electrical recovery phase characterized by a reduction in the ST segment elevation and improvement of the local activation delay with reappearance of local intrinsic deflections on previously monophasic potentials (Supplementary Figure S3). This phase began at about 45–60 min of occlusion and ended 30 min thereafter. In all animals, we observed a correspondence between the anatomopathological substrate and the electrical mapping. This correspondence was established by identifying first the necrotic regions in the serial section of the atria stained with Masson’s trichrome and then determining their projection into the 128-electrode patch, which was marked with Prolene snares and photographed at the end of the experiment. Necrotic regions corresponded to electrode areas with monophasic potentials, and healthy regions depicted normal electrograms (Figure 1, bottom panel).

The transition between electrodes with ischemic changes and electrodes with normal potentials encompassed less than two contiguous electrode sites (Figure 4B). Thus, since the mapping electrodes were spaced 1 mm, the expected width of the electrical border zone would be smaller than 2 mm. The area of the epicardial mapping encompassing electrodes with monophasic potentials increased during the occlusion period (Figure 4B). The number of electrodes showing monophasic potentials increased overtime (28 ± 35 at 60 min, 98 ± 37 at 120 min, and 112 ± 18 at 240 min; *p* < 0.01).

### Surface ECG Findings

**FIGURE 5:**
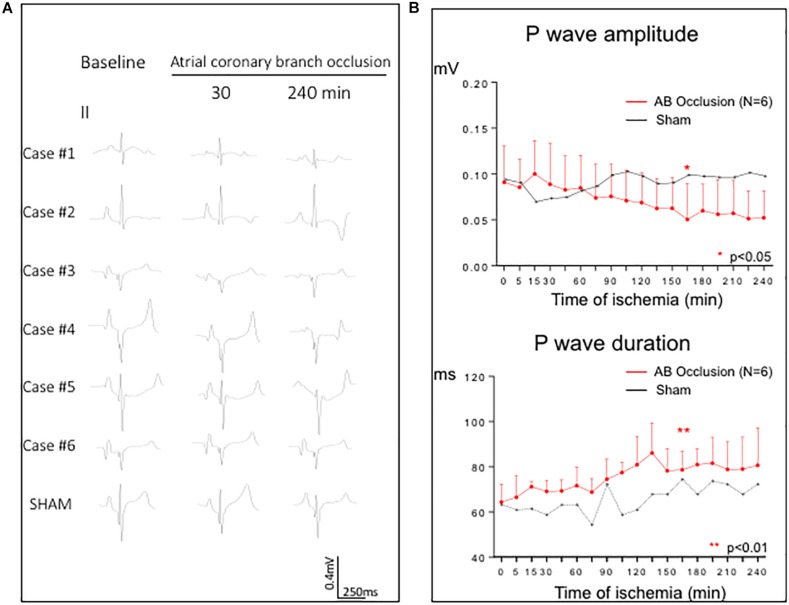
Changes in P wave morphology induced by 4 h of atrial coronary branch occlusion. **(A)** ECG lead II for each of the six infarcted pigs at baseline and after 30 and 240 min of atrial coronary branch occlusion. The bottom row shows the absence of P wave changes in a pig not submitted to coronary occlusion (sham experiment). **(B)** Time course of the P wave mean amplitude and duration in the entire group of the six pigs submitted to 4 h of atrial branch occlusion (red line). The plateau behavior of the P wave amplitude and duration in one sham pig is represented by black line. AB, atrial coronary branch. *p < 0.05, **p < 0.01; from 0 to 165 min of ischemia.

As shown in Figure 5, changes in P wave morphology were only appreciable late after 2 h of coronary occlusion. After 165 min of occlusion P wave amplitude decreased from 0.09 ± 0.04 mV at baseline to 0.05 ± 0.04 mV (*p* < 0.05) and P wave duration increased from 64.4 ± 8.0 ms to 80.9 ± 12.6 ms (*p* < 0.01). We did not observe appreciable deviations of the PR-segment level in any ECG lead. The sham pig did not show noticeable changes in the P wave morphology. Selective occlusion of the atrial branch did not induce concurrent ventricular ischemia as denoted by the absence of significant changes in the QRS-complex duration and ST segment level (Supplementary Figure S4). Episodes of atrial fibrillation were not observed.

## Discussion

### Main Findings

This study is the first describing the changes in local atrial electrograms induced by acute occlusion of an atrial coronary branch and their temporal relationship with the ECG. Using a model close to the human cardiac anatomy and electrophysiology (Cinca et al., 1980), we have found that this procedure induces patchy atrial infarction with sharp border zone and evolving changes of atrial R/S waves, ST segment, and local activation time. The early ischemic changes in atrial electrograms were not detectable on the ECG, and only variations in P wave morphology were appreciated in later stages of atrial branch occlusion.

### Anatomopathological Findings

The structural features of atrial infarction have been analyzed in necropsy studies of patients who presented a concomitant ventricular myocardial infarction caused by occlusion of the RCA or LCX (Cushing et al., 1942; Liu et al., 1961). In these studies, the atrial infarction was massive, suggesting that the interruption of coronary flow affected most of the atrial branches that ordinarily arise from the proximal segment of the RCA or LCX (Baroldi et al., 1956; James and Burch, 1958). Similarly, proximal LCX occlusion induced large atrial infarcts and atrial dilation in pigs (Aguero et al., 2017), which, like humans, have a comparable emergence of the atrial branches from the RCA or LCX coronary arteries (Weaver et al., 1986). By contrast, the structural changes induced by selective atrial branch occlusion have been only described by a single research group in dogs and sheep (Sinno et al., 2003; Rivard et al., 2007; Avula et al., 2018). In dogs, the isolated occlusion of an atrial branch of the RCA induced atrial ischemic changes, which were often nearly transmural at the center of the ischemic area but followed a patchy distribution at the periphery. Similar to previous studies in dogs (Sinno et al., 2003; Rivard et al., 2007), our study in pigs showed that a selective occlusion of an atrial branch of the RCA induced a patchy atrial necrosis pattern, perhaps favored by coronary collaterals that ordinarily interconnect the atrial branches in human and porcine hearts (Baroldi et al., 1956; James and Burch, 1958; Weaver et al., 1986). Bragt et al. (2015) measured the coronary blood flow through an atrial branch of the LCX coronary in pigs and found that it was maximal in the early diastolic phase and ranged 5–10 ml/min.

### Epicardial Atrial Mapping

This study reveals that during the first 30 min of selective atrial branch occlusion, the local atrial electrograms show a progressive increase in R wave amplitude associated with parallel prolongation of the local activation time and elevation of the ST segment leading to monophasic potentials. These changes are fully comparable to those described in acute ventricular myocardial ischemia (Janse et al., 1979; Cinca et al., 1980), and this resemblance suggests that atrial and ventricular myocardial ischemia share similar cellular electrophysiological derangements. Assuming this parallelism, the R/S wave changes would indicate the existence of a slow conduction in the ischemic atrial myocardium and the ST segment elevation from a flow of injury currents created by the loss of resting membrane potential of ischemic cells (Kléber et al., 1978). Likewise, monophasic potentials will indicate local atrial cellular inexcitability. In two cases, we observed a transient recovery of local activation time and a reduction in the ST segment elevation similarly found in acute ventricular myocardial ischemia, also in swine. This is not due to a concurrent structural improvement of the ischemic myocardium but rather to a concurrent transitory decrease in potassium accumulation in the ischemic myocardium (Janse et al., 1979; Coronel et al., 2019).

One hour after atrial branch occlusion, there was a progressive decrease in the magnitude of ST segment elevation despite the persistence of the coronary clamping. The decline of the ST segment elevation overtime was constantly observed in ventricular myocardial ischemia and is caused by the progressive increase in the electrical resistivity in the ischemic myocardium, which impedes the propagation of the injury currents throughout the ischemic and normal regions (Kléber et al., 1987; Cinca et al., 1997).

The electrical border zone of atrial infarction was abrupt. The atrial mapping showed that the transition between electrodes with normal electrograms and electrodes with ST segment elevation was about 1–2 mm. Of note, this electrical border shifted during the course of ischemia as denoted by the progressive increase in the number of electrodes showing monophasic potentials after 2 h of atrial branch occlusion. These changes have not been appreciated in ventricular myocardial ischemia in the same experimental model (Janse et al., 1979). Reasons for the time-dependent instability of the atrial area at risk cannot be fully elucidated. However, local edema induced in the course of acute myocardial ischemia could interfere atrial coronary perfusion and expand the injured area. In favor of the presence of local edema, we found a reduction in the amplitude of local electrogram that have not shown ischemic ST segment changes.

Atrial arrhythmias, and particularly atrial fibrillation, have been considered diagnostic clues to suspect atrial infarction involvement in patients with acute occlusion of the RCA or LCX coronary arteries (Lazar et al., 1988; Lu et al., 2016). Atrial epicardial mapping in dogs with selective atrial branch occlusion showed slowing of atrial conduction and prolongation of the refractory period, leading to a substrate for atrial fibrillation maintenance (Sinno et al., 2003). However, these arrhythmias were absent in our study and also in dogs, in which only increased stability of electrically induced atrial fibrillation was attained (Sinno et al., 2003).

### ECG Findings

This study allows to understand the limited diagnostic value of the surface ECG to recognize atrial infarction. The acute ischemic changes in the atrial R/S waves and local activation times translate to the surface ECG as unspecific variations of P wave morphology, but these were only appreciable at advanced stages of atrial coronary occlusion. On the other hand, the characteristic ischemic displacements of the atrial ST segment did not induce appreciable shifts of the PR segment at the surface ECG. A relatively low incidence (20%) of PR-segment displacement was reported in a series of 39 patients with acute inferior myocardial infarction submitted to primary coronary angioplasty and angiographic documentation of atrial branch involvement (Yıldız et al., 2018). Moreover, the PR-segment deviation can vanish over time as was reported in a prospective series of 17 patients with accidental occlusion of atrial branch during elective coronary angioplasty (Álvarez-García et al., 2016).

Selective atrial branch occlusion did not elicit ST segment changes in the surface ECG, thus confirming the absence of concurrent ventricular myocardial ischemia in our atrial infarction model.

### Study Limitations

A median sternotomy was needed to perform the atrial epicardial mapping and the direct ligature of an atrial coronary branch. Since this intervention modifies the spatial relationship of the surface ECG leads and the heart, we focused our analysis on lead II because it was the less affected by the sternotomy and is currently accepted as an appropriate lead to evaluate the P wave morphology. The relatively small sample size of this study only allows a qualitative description of the characteristics and time course of the local atrial electrograms induced by selective acute atrial infarction. This proof-of-concept approach affords foundation for future, more highly powered studies aimed at describing ECG markers of the extension and location of the infarction in the right or left atrial chambers.

This study does not allow determining the influence of the extent of atrial infarction on the magnitude of the P wave changes of the surface ECG. The median sternotomy needed to perform both the atrial mapping and the direct ligature of the coronary atrial branches disrupts the spatial relationship of the heart with the ECG leads and, consequently will hamper any attempt to accurately correlate the magnitude of the P wave changes with the size of atrial infarction. However, the finding of significant changes in the P wave morphology during atrial ischemia in our study suggests, at least, that atrial infarction was large enough to project into the surface ECG. Future studies in closed chest models are therefore needed to precisely quantify the effects of atrial infarct size on the P wave characteristics. The lack of quantification of the atrial infarct size in this study does not invalidate the epicardial mapping data because the changes in local atrial electrograms are not dependent on the remote extension of infarction but only on local site-to-site transmural spanning of the ischemic injury.

### Clinical Implications

Translation of our findings to the clinical setting is tenable because the distribution of atrial coronary branches and the electrophysiological derangements caused by acute myocardial ischemia in pigs and humans are comparable (Weaver et al., 1986; Cinca et al., 1998).

Our study suggests that although acute occlusion of an atrial branch in patients with coronary atherosclerosis would cause atrial infarction, this condition would be hardly recognized by the surface ECG. Indeed, the specific acute local electrical alterations were only reflected by unspecific changes in P wave morphology at late stages of occlusion. However, acute atrial myocardial ischemia would be reliably detected clinically by direct atrial recordings, and this may open new diagnostic technological developments applicable to patients with implanted electrostimulation devices.

## Data Availability Statement

The datasets generated for this study are available on request to the corresponding author.

## Ethics Statement

The animal study was reviewed and approved by the Animal Experimentation Ethics Committee Research Institute of Hospital de la Santa Creu i Sant Pau.

## Author Contributions

GA-F and JC made substantial contributions to the conception and design of the work, participated in the acquisition, analysis, and interpretation of the data, drafted the manuscript, and given the final approval of the version to be published and the agreement to be accountable of all aspects of the work in ensuring that questions related to the accuracy or integrity of any part of the work are appropriately investigated and resolved. ER-D, DS-Q, SC-R, EJ, JN-M, DA, XM, CA-M, and JG made substantial contributions to the design of the work, participated in the acquisition of the data, revised the draft critically for important intellectual content, and given the final approval of the version to be published and the agreement to be accountable of all aspects of the work in ensuring that questions related to the accuracy or integrity of any part of the work are appropriately investigated and resolved.

## Conflict of Interest

The authors declare that the research was conducted in the absence of any commercial or financial relationships that could be construed as a potential conflict of interest.

## Supplementary Material

FIGURE S1 Photographs illustrating the correspondence between the corners of the 128-patch electrode **(A)** and the fixed black Prolene snare marks **(B)**.

FIGURE S2 Representative microphotographs illustrating the morphometric analysis of the extent of area of myocardial myolysis. **(A–C)** show examples of areas of myolysis of 38, 46, and 72%, respectively.

FIGURE S3 Evolving changes in epicardial atrial electrograms in a pig submitted to 4-h of atrial coronary branch occlusion showing a transient recovery of local activation between 30–60 min of occlusion.

FIGURE S4 Graphs showing the stability of the mean values of ventricular ST-segment and QRS duration at baseline and at specific times of atrial branch occlusion measured in lead II in six pigs submitted to 4-h of atrial coronary branch occlusion.
