# Supplementary figures and images for: Changes in Local Atrial Electrograms and Surface ECG Induced by Acute Atrial Myocardial Infarction
Source: Front Physiol. 2020 Apr 17;11:264. doi: 10.3389/fphys.2020.00264 (PMC7180211; doi:10.3389/fphys.2020.00264)

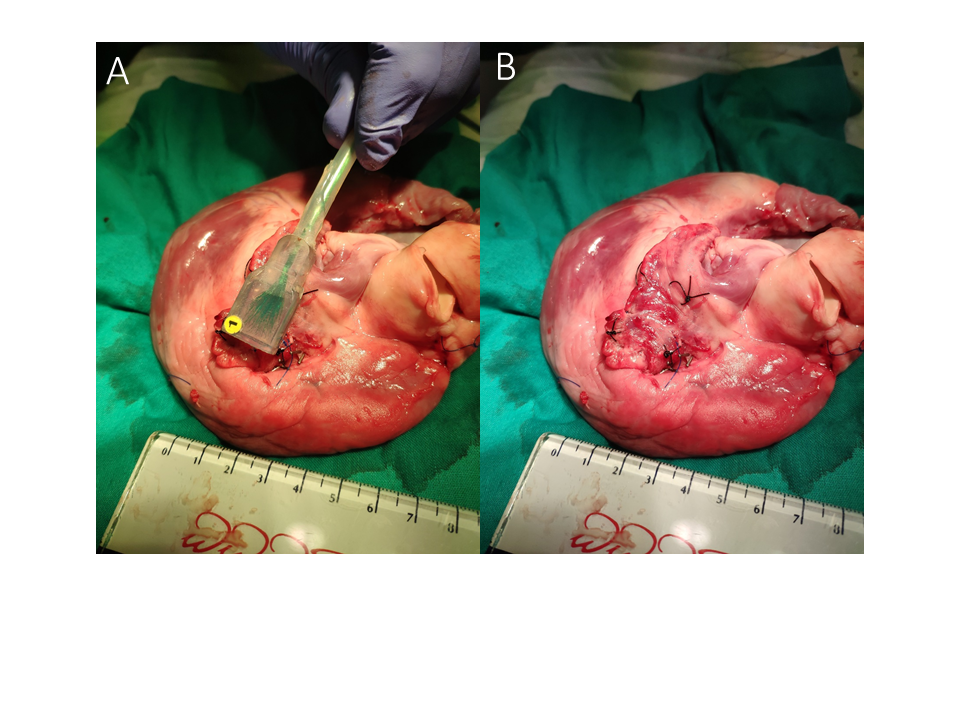

Supplement: FIGURE S1 — Photographs illustrating the correspondence between the corners of the 128-patch electrode (A) and the fixed black Prolene snare marks (B). [file Image_1.TIF]

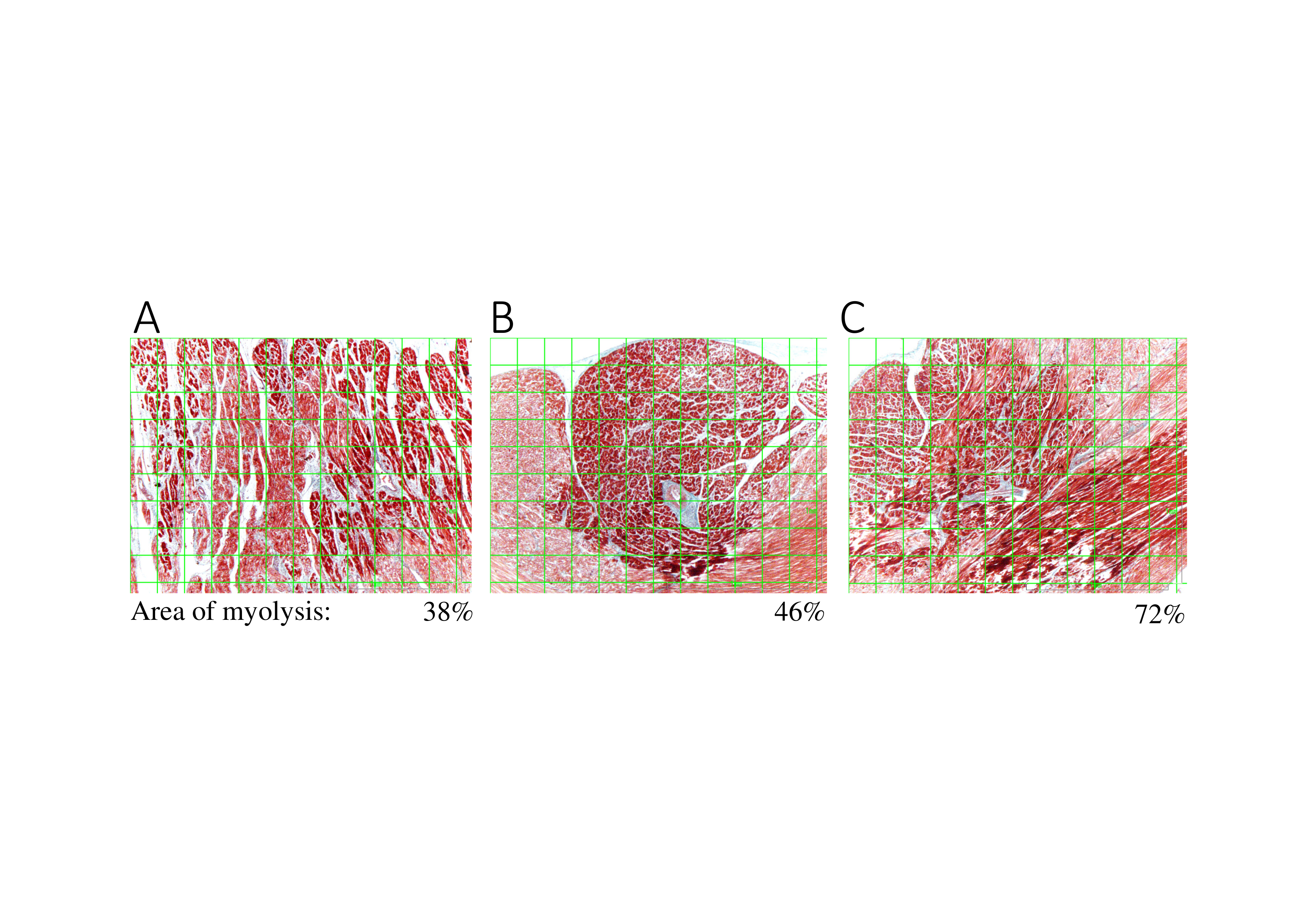

Supplement: FIGURE S2 — Representative microphotographs illustrating the morphometric analysis of the extent of area of myocardial myolysis. (A–C) show examples of areas of myolysis of 38, 46, and 72%, respectively. [file Image_2.TIF]

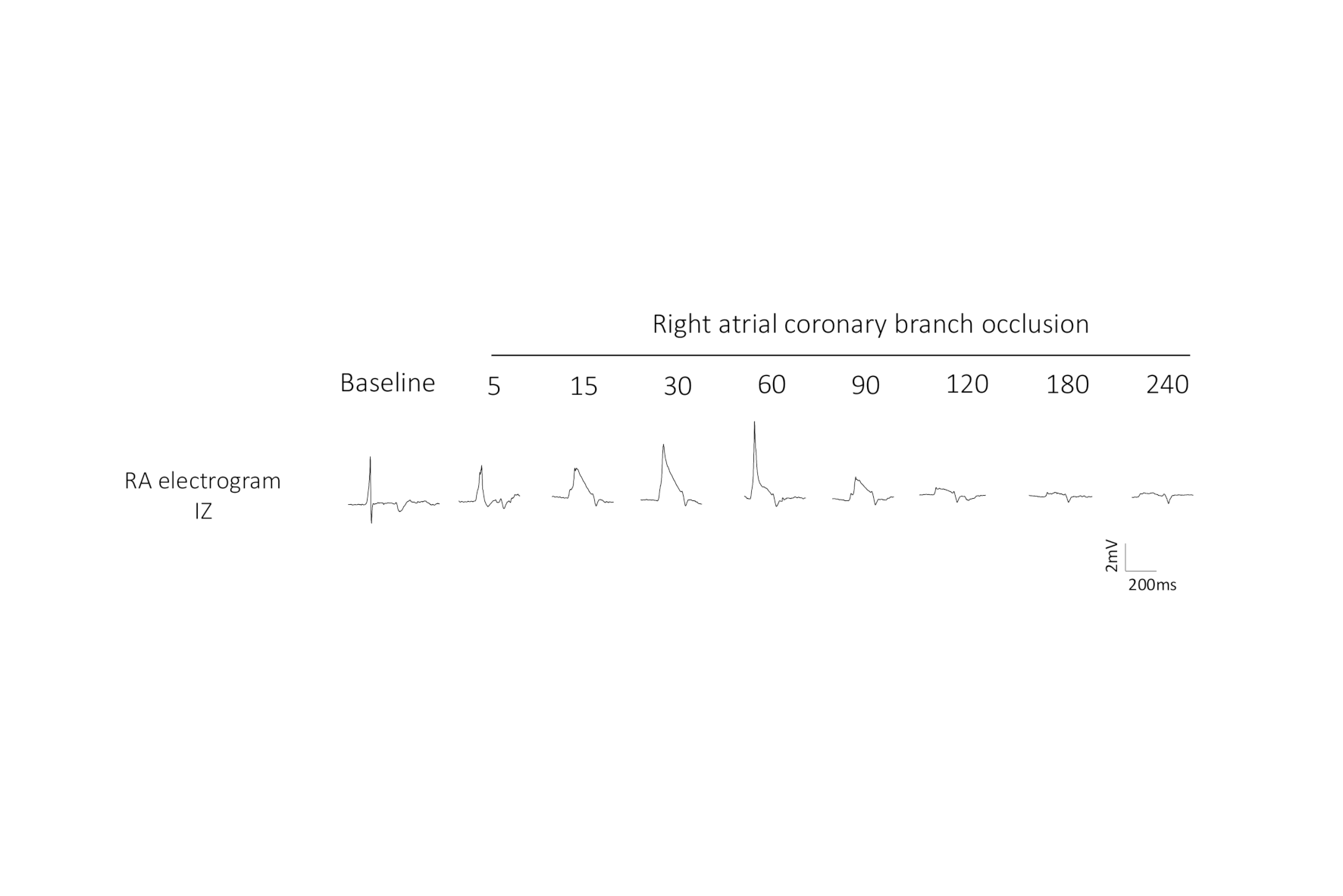

Supplement: FIGURE S3 — Evolving changes in epicardial atrial electrograms in a pig submitted to 4-h of atrial coronary branch occlusion showing a transient recovery of local activation between 30–60 min of occlusion. [file Image_3.TIF]

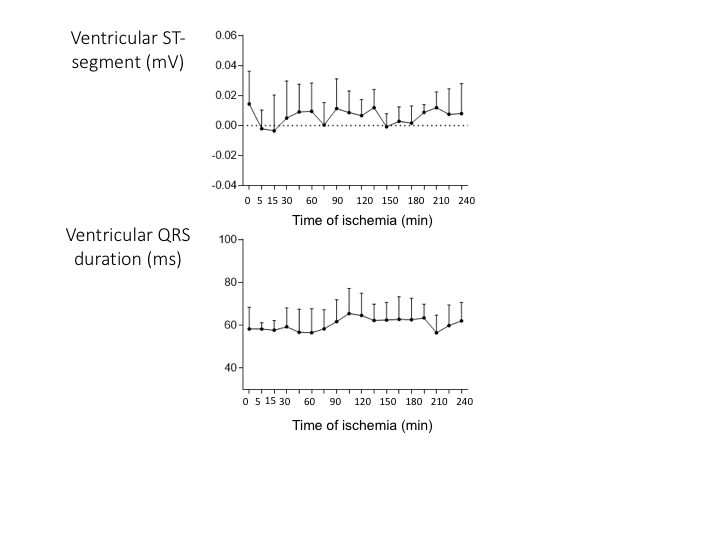

Supplement: FIGURE S4 — Graphs showing the stability of the mean values of ventricular ST-segment and QRS duration at baseline and at specific times of atrial branch occlusion measured in lead II in six pigs submitted to 4-h of atrial coronary branch occlusion. [file Image_4.TIFF]
